# Pilot trial using mass field-releases of sterile males produced with the incompatible and sterile insect techniques as part of integrated *Aedes aegypti* control in Mexico

**DOI:** 10.1371/journal.pntd.0010324

**Published:** 2022-04-26

**Authors:** Abdiel Martín-Park, Azael Che-Mendoza, Yamili Contreras-Perera, Silvia Pérez-Carrillo, Henry Puerta-Guardo, Josué Villegas-Chim, Guillermo Guillermo-May, Anuar Medina-Barreiro, Hugo Delfín-González, Rosa Méndez-Vales, Santos Vázquez-Narvaez, Jorge Palacio-Vargas, Fabián Correa-Morales, Guadalupe Ayora-Talavera, Norma Pavía-Ruz, Xiao Liang, Ping Fu, Dongjing Zhang, Xiaohua Wang, María Eugenia Toledo-Romaní, Zhiyong Xi, Gonzalo Vázquez-Prokopec, Pablo Manrique-Saide

**Affiliations:** 1 Laboratorio para el Control Biológico de *Aedes aegypti* (LCB-UADY), Unidad Colaborativa para Bioensayos Entomológicos, Campus de Ciencias Biológicas y Agropecuarias, Universidad Autónoma de Yucatán, Mérida, México; 2 Servicios de Salud de Yucatán, Mérida, México; 3 Centro Nacional de Programas Preventivos y Control de Enfermedades (CENAPRECE), Secretaría de Salud, Ciudad de México, México; 4 Centro de Investigaciones Regionales “Dr. Hideyo Noguchi”, Universidad Autónoma de Yucatán, Mérida, México; 5 Department of Microbiology and Molecular Genetics, Michigan State University, East Lansing, Michigan, United States of America; 6 School of Basic Medical Sciences, Guizhou Medical University, Guiyang, China; 7 Sun Yat-sen University–Michigan State University Joint Center of Vector Control for Tropical Diseases, Guangzhou, China; 8 Guangzhou Wolbaki Biotech Co. Ltd., Guangzhou, Guangdong, China; 9 Departamento de Epidemiología, Instituto de Medicina Tropical "Pedro Kourí" (IPK), La Habana, Cuba; 10 Department of Environmental Sciences, Emory University, Atlanta, Georgia, United States of America; Liverpool School of Tropical Medicine, UNITED KINGDOM

## Abstract

**Background:**

The combination of *Wolbachia*-based incompatible insect technique (IIT) and radiation-based sterile insect technique (SIT) can be used for population suppression of *Aedes aegypti*. Our main objective was to evaluate whether open-field mass-releases of *w*AlbB-infected *Ae*. *aegypti* males, as part of an Integrated Vector Management (IVM) plan led by the Mexican Ministry of Health, could suppress natural populations of *Ae*. *aegypti* in urbanized settings in south Mexico.

**Methodology/Principal findings:**

We implemented a controlled before-and-after quasi-experimental study in two suburban localities of Yucatan (Mexico): San Pedro Chimay (SPC), which received IIT-SIT, and San Antonio Tahdzibichén used as control. Release of *w*AlbB *Ae*. *aegypti* males at SPC extended for 6 months (July-December 2019), covering the period of higher *Ae*. *aegypti* abundance. Entomological indicators included egg hatching rates and outdoor/indoor adult females collected at the release and control sites. Approximately 1,270,000 lab-produced *w*AlbB-infected *Ae*. *aegypti* males were released in the 50-ha treatment area (2,000 *w*AlbB *Ae*. *aegypti* males per hectare twice a week in two different release days, totaling 200,000 male mosquitoes per week). The efficacy of IIT-SIT in suppressing indoor female *Ae*. *aegypti* density (quantified from a generalized linear mixed model showing a statistically significant reduction in treatment versus control areas) was 90.9% a month after initiation of the suppression phase, 47.7% two months after (when number of released males was reduced in 50% to match local abundance), 61.4% four months after (when initial number of released males was re-established), 88.4% five months after and 89.4% at six months after the initiation of the suppression phase. A proportional, but lower, reduction in outdoor female *Ae*. *aegypti* was also quantified (range, 50.0–75.2% suppression).

**Conclusions/Significance:**

Our study, the first open-field pilot implementation of *Wolbachia* IIT-SIT in Mexico and Latin-America, confirms that inundative male releases can significantly reduce natural populations of *Ae*. *aegypti*. More importantly, we present successful pilot results of the integration of *Wolbachia* IIT-SIT within a IVM plan implemented by Ministry of Health personnel.

## Author summary

Wild-type female *Ae*. *aegypti* mating with released males carrying the maternally inherited bacteria *Wolbachia* produce infertile eggs, leading to important reductions in mosquito population size. We present results from pilot open-field mass-releases of *Ae*. *aegypti* males infected with the *Wolbachia* strain *w*AlbB (termed incompatible insect technique, IIT) and irradiated to prevent accidental female mosquito colonization (termed sterile insect technique, SIT). Our IIT-SIT approach was implemented by the Mexican Ministry of Health within an Integrated Vector Management (IVM) plan to suppress natural populations of *Ae*. *aegypti*. Approximately 1,270,000 lab-produced *w*AlbB-infected *Ae*. *aegypti* males were released in a 50-ha. town of Yucatan over a period of 24 weeks. Throughout the suppression phase, we observed significant reductions in egg hatching, outdoor and indoor female *Ae*. *aegypti* densities in the release town compared to a similar town used as control. The largest effect was on the number of indoor *Ae*. *aegypti* females per house (Prokopack collections) which reached a 90% efficacy. Our study, the first report of an open-field pilot-study with mass-releases of sterile *Ae*. *aegypti* males produced with IIT-SIT in Mexico and Latin-America, confirms findings from other settings showing important reductions in entomological indices due to inundative incompatible male releases.

## Introduction

A revolution in the control of *Aedes* vectors of arboviral diseases is spearheaded by the release of *Wolbachia*-transinfected mosquitoes [1–6]. Specifically, the endosymbiont bacterium *Wolbachia* is being used as a biological control agent of the mosquito *Aedes aegypti*, the main vector of dengue, chikungunya and Zika, via incompatible mating (population suppression) and/or by inhibiting viral multiplication within mosquitoes (population replacement) [7–9]. For population suppression, some *Wolbachia* strains carry a cytoplasmic incompatibility phenotype, where males with a particular *Wolbachia* strain can be reproductively incompatible with females that do not have the same strain, which causes early embryonic arrest and egg hatch failure [10]. For population replacement, *Wolbachia* strains cause different levels of pathogen interference as *Wolbachia*-infected female mosquitoes are less susceptible to transmit dengue (DENV), chikungunya (CHIKV) and Zika (ZIKV) viruses [11–13].

All approaches for *Wolbachia* biocontrol involve operational strategies based in the “rear and release” of *Ae*. *aegypti* as a *“*self-delivering” control method [14]. Population suppression requires large (mass) field-releases of sterile males carrying *Wolbachia* to induce incompatible mating with wild females, while population replacement requires more “limited” (smaller) releases of infected males and females that will eventually establish the bacterium in the wild population [7,14]. Field-releases of *Wolbachia*-infected *Ae*. *aegypti* for both approaches are under implementation in different countries [1–3,5,15–22]. The population suppression approach through *Wolbachia*-induced cytoplasmic incompatibility (CI) (known as IIT or incompatible insect technique) uses a *Wolbachia* strain (*w*AlbB) from *Ae*. *albopictus* (donor host) that has been successfully established in *Ae*. *aegypti* (novel host) [3,6,10]. The population replacement approach has been using primarily *w*Mel (from *Drosophila*) and less commonly *w*AlbB to interrupt transmission of DENV in *Wolbachia*-infected mosquitoes [2,4,15,23].

In Mexico, both population replacement and suppression are under an initial phase of implementation and evaluation for the control of *Ae*. *aegypti*, with *w*Mel being piloted in Baja California (North Mexico) [24] and *w*AlbB in Yucatan (South Mexico) [25], respectively. In 2016, the government of the Mexican state of Yucatan signed an international collaboration agreement with the Autonomous University of Yucatan (UADY) and Michigan State University (MSU) for the development and application of *Wolbachia*-based strategies to suppress *Ae*. *aegypti* populations. The Laboratory of Biological Control for *Aedes aegypti* (LCB-UADY) has an installed capacity to produce 1–5 million sterile *Ae*. *aegypti* males per week [25] with premises, equipment and production processes following, at a lower scale, those established by MSU and Sun Yat-sen University Joint Center of Vector Control for Tropical Diseases in Guangzhou, China [1,26–28].

The LCB-UADY combines the insect incompatible technique through *Wolbachia*-induced incompatibility with the sterile insect technique using radiation (known as IIT-SIT) [1] and produces irradiated mass-reared adult *Ae*. *aegypti* males of a local mosquito line infected with *w*AlbB. Due to CI, wild-type female *Ae*. *aegypti* mating with released males carrying the maternally inherited *Wolbachia* will produce infertile eggs which leads to subsequent reductions in adult mosquito populations [7,9,10]. The IIT-SIT method was of interest by Yucatan because it involved releases of male mosquitoes *Aedes* (males do not bite, blood feed, or transmit disease-causing pathogens to humans) for population suppression. After sex separation and irradiation, any *Wolbachia*-carrying female accidentally released would not be able to reproduce, minimizing any undesirable establishment of *Wolbachia* in the wild. IIT-SIT demonstrated successful implementation to control *Ae*. *albopictus* in field pilot tests in China [1], and suppression of natural populations of *Ae*. *aegypti* in Thailand [17] and Singapore [3]. From a more technical perspective, the *w*AlbB strain of *Wolbachia* has stable and strong CI in *Ae*. *aegypti* with minimal effects on mosquito fitness and male mating success [10,29], with well stability/persistent in the mosquito against areas/regions with high temperatures [30] which makes it a suitable option for population replacement/suppression in warm climates [23], like Yucatan.

The release model in Yucatan is led by the Ministry of Health (MoH), with LCB-UADY overseeing mosquito rearing, intervention, and evaluation. In 2019, the local MoH carried-out a pilot test to implement IIT-SIT as part of integrated *Ae*. *aegypti* control at the municipality of Merida Yucatan. LCB-UADY designed an Integrated Vector Management (IVM) plan, in coordination with the Yucatan MoH [25], which included routine vector control activities [31] and the field-release of *Ae*. *aegypti w*AlbB males to suppress a local mosquito population. Here we report the results of the implementation of this IVM plan incorporating IIT-SIT for the control of *Ae*. *aegypti* in a suburban locality of Merida, Mexico. Our study represents the first open field release of *Ae*. *aegypti* males developed from a combined IIT-SIT approach in Mexico and Latin-America.

## Methods

### Ethics statement

The pilot suppression trial of *Ae*. *aegypti* using *Wolbachia* IIT-SIT was reviewed and approved by the Review Board of the Campus of Biological, Veterinary and Agricultural Sciences of UADY (CB-CCBA-I-2019-005). The open field mosquito releases were approved by the government and the health authorities of Yucatan. All the activities of the vector control program remained under normal operation in both release and untreated control areas.

### Study sites and design

**Fig 1:**
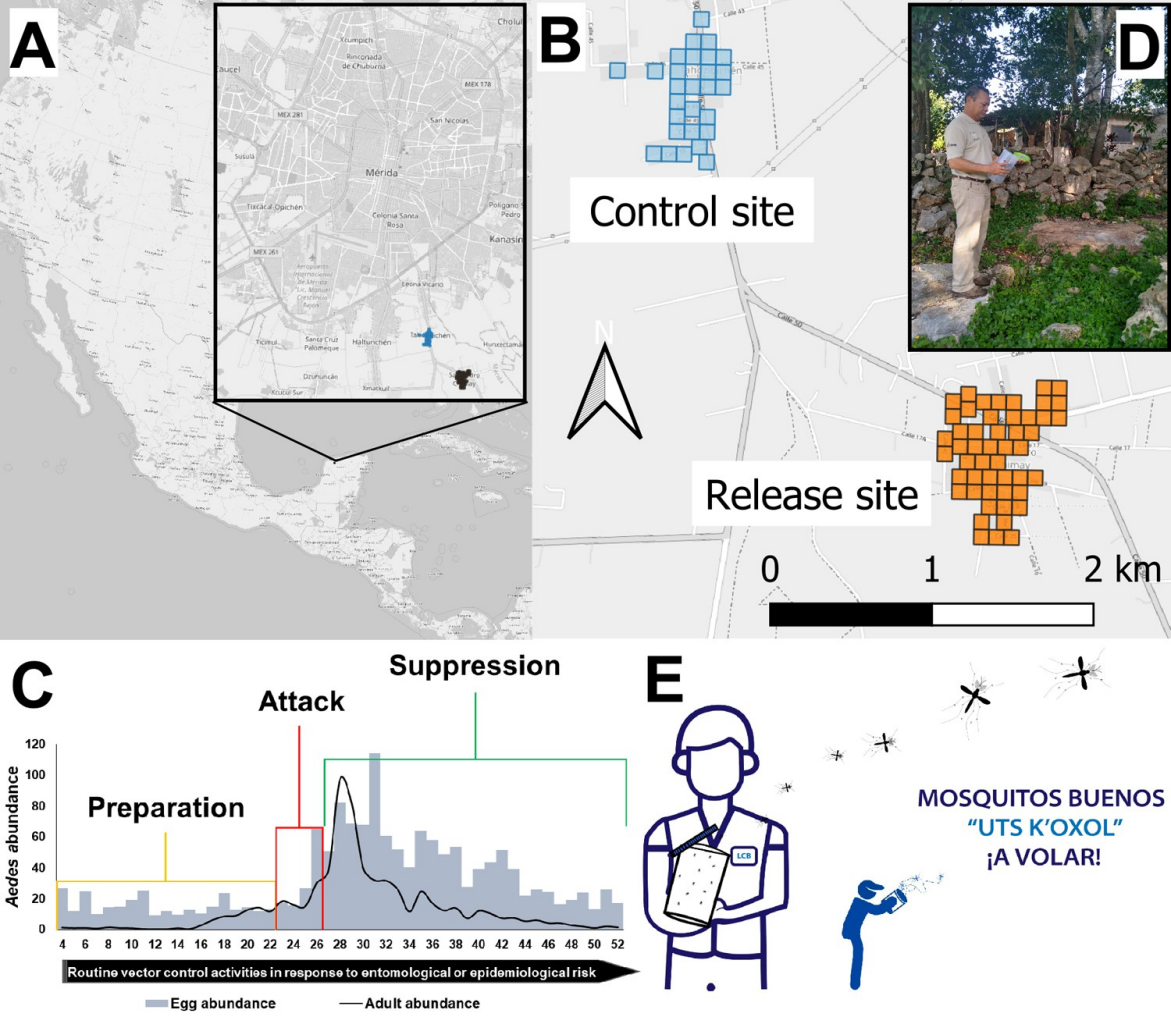
A) Field sites located in the periphery of the city of Merida, southeast of Mexico; San Pedro Chimay (release site) shown in orange and Tahdzibichén (control site) in blue; B) Distribution of one-hectare areas within localities, where sentinel areas for entomological surveillance were located and releases were performed (only at San Pedro Chimay); C) Proposed an Integrated Vector Management plan combining ‘traditional *Aedes* control’ and the release of X-ray irradiated male *Ae*. *aegypti* carrying *Wolbachia* for population suppression structured in three phases: Preparation: with community sensitization and engagement, and baseline entomological studies; Attack: initial traditional vector control; and Suppression phase: with inundative releases during peak of mosquito abundance; D) Sterile male mosquitoes released in a backyard by personnel of the MoH; E) "Uts koxol” (“good mosquitoes” in Mayan language). Maps were produced using QGIS based on public geographic data obtained from OpenStreetMap (www.openstreetmap.org).

The study sites were San Pedro Chimay (hereafter SPC) and San Antonio Tahdzibichén (hereafter TAH), two comparable suburban localities in the periphery of the city of Merida (Fig 1A). Localities, selected in consensus with the MoH of Yucatan, have a predefined set of entomological, ecological, sociological, and logistical inclusion criteria recommended for a Phase II (the attack phase included routine vector control measures to lower, at both localities, mosquito populations prior to the mass-release of *w*AlbB *Ae*. *aegypti*) study *v*.*gr*. a pilot or initial implementation study [25,32–34]. A controlled, before-and-after control-intervention (BACI) quasi-experimental study design was developed following the design of other area-wide IIT-SIT interventions [1,17], with one locality randomly assigned to receive the IIT-SIT intervention/release (SPC) and the other considered as control (TAH).

Both localities are small suburban towns (30–50 ha), separated from each other and from large urban centers but sharing similar environments. The release site SPC has a population of 1,241 individuals across 300 houses (50 ha) (Fig 1A and 1B). The control site TAH has a population of 724 individuals across 174 houses (30 ha). They are 2.5 km apart, surrounded by local vegetation (disturbed tropical dry forest). The average elevation of the localities is nine meters above sea level. The climate is tropical with an annual average temperature of 26.3°C (34.2°C max- 18.4°C min), with two distinct phases in a year: a rainy season, from May/June to October with a rainfall of 990.6 mm; and a dry season from November to April with rainfall of 291.2 mm [35].

An extensive description of the baseline information gathered during a former Phase I has been published [25] and at both study sites, *Ae*. *aegypti* abundance/density and population dynamics were similar [25]. The rainy season leads to the peak of *Ae*. *aegypti* population abundances in the study sites [25]. At both localities, *Ae*. *aegypti* uses outdoor breeding sites including water drums (metal or plastic), bowls for feeding animals, cooking, and washing utensils, and plant pots. *Aedes albopictus* was not encountered before the study. None of the localities had ongoing reports of *Aedes* Borne Diseases (ABD) cases during the time of the study.

### Integrated vector management plan using routine vector control and field releases of sterile males produced with IIT-SIT

**Fig 2:**
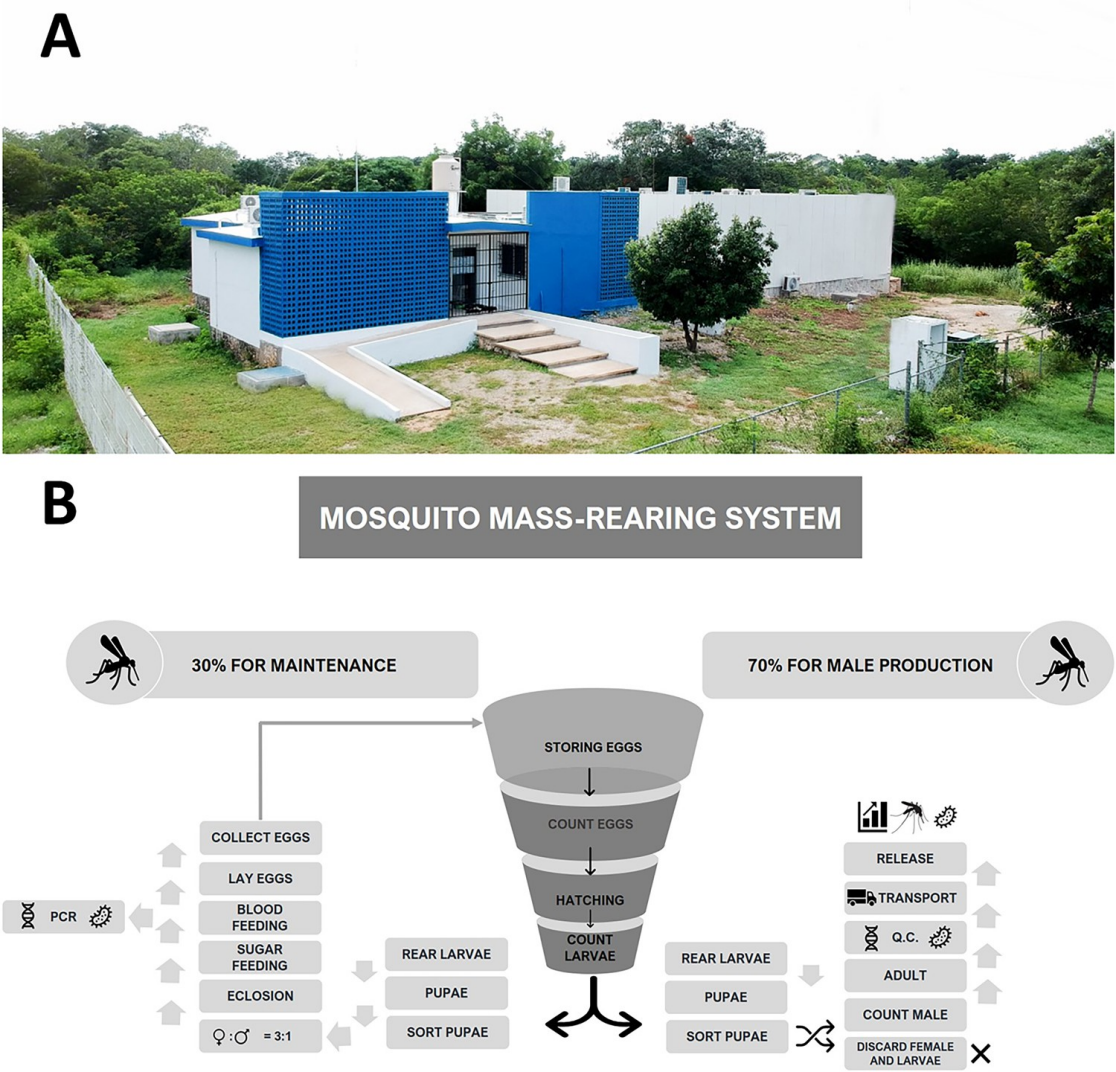
A) Laboratory of Biological Control for *Aedes aegypti* (LCB-UADY). B) Schematic representation of the main processes at LCB-UADY to produce *w*AlbB *Ae*. *aegypti* mosquitoes with the sterile insect technique using radiation (IIT-SIT). As part of the Q.C., we optimized a PCR assay to detect the presence of *Wolbachia* infection in every generation of adult mosquitoes under mass-rearing system as well as before every release.

Details of our IIT-SIT integration within an IVM plan were described in Che-Mendoza et al. [25] and included three phases: 1) Preparation: with social work for community sensitization and engagement and baseline entomological studies; 2) Attack: vector control with routinary activities performed by the MoH; and 3) Suppression: with inundative releases of male mosquitoes produced at the LCB-UADY (Fig 2A) during the peak of abundance of *Ae*. *aegypti* populations.

### Preparation phase

#### Social work

We developed a plan for social integration and support of the community during the different stages of the project, following existing World Health Organization (WHO) recommendations and prior studies [1,17,33,36]. A social team involving Collaborative Unit for Entomological Bioassays (UCBE) and LCB-UADY project staff in collaboration with MoH personnel, implemented the social engagement plan (Table A in S1 Appendix). A strategic element of the project was the acceptance and engagement of local authorities and stakeholders, in addition to community/families. Another major goal of our social work was understanding the perception of the community and the barriers for mass-releases of male *Ae*. *aegypti* mosquitoes. The community leaders initially recognized the paradigm shift of “killing mosquitoes versus releasing mosquitoes” as a barrier to the implementation. However, the explanation that males do not bite, that they do not blood-feed and that cannot transmit diseases, and that male-releases were part of an IVM approach in synergy -with known and accepted routine methods- to reduce female mosquitoes was a facilitator. 95% of participants were willing to accept the release of male mosquitoes, and they had a good social reception to the vector control program run by MoH of Yucatan. The “identity of the project” was an important element promoting the use of mass-releases of *w*AlbB *Ae*. *aegypti* males. We adopted the project slogan "Uts k’oxol” (“good mosquitoes” in the Mayan language) (Fig 1E) considering the sociocultural context of the study area (predominance of Mayan roots in residents), which gave a sense of belonging and facilitated adoption of the intervention by the community. In preparation for mosquito releases, and in agreement with householders, we selected release spots (one per city block, see below) within backyards of houses. Families that accepted to become part of the component were informed about the release schedule and were provided with extra educational material to reinforce the knowledge of the intervention, becoming local promoters of the benefits and achievements of the project.

#### Pre-release entomological survey

Pre-intervention monitoring of *Ae*. *aegypti* populations at both localities started two years prior releases (2017–2018). Results have been published [25] and were used to: a) confirm suitability of study localities; b) develop the IVM approach incorporating IIT-SIT (see below); c) calculate likely release ratios throughout the season; d) confirm feasibility of our production system at the LCB-UADY. Entomological surveys were conducted in one-hectare areas (Fig 1B, and Che-Mendoza et al. [25]) that were monitored weekly with two ovitraps/ha (100 per locality) and 24 h- weekly collections with one BG sentinel trap (Biogents, baited with octenol (1-octen-3-ol 55.15%, Mosquito Magnet Octenol Attractant) per hectare. Indoor adult mosquito collections were performed prior to the intervention with Prokopack aspirators [37] for a 10-min period per house in a sub-sample of houses sampled with BG traps.

### Attack phase with traditional vector control

The *Aedes* vector control program of Yucatan [31] routinely organizes and implements clean up campaigns for breeding-sites disposal (*descacharrización* in Spanish) before the rainy season. Truck-mounted ultra-low volume (ULV) application of adulticides from a list of approved products [38] is also performed in response to increased risk of arbovirus transmission, suggested by mosquito abundance. The program additionally performs indoor space spraying and truck mounted ULV in reaction to reported cases of arboviral disease [31]. With this toolbox, we developed an IVM strategy incorporating IIT-SIT to routine control activities in coordination with the MoH [25] (Fig 1C). About insecticide susceptibility/resistance, recent studies have shown that *Ae*. *aegypti* wild populations are resistant to multiple pyrethroids in Merida, with evidence of knockdown resistance (kdr) mutations (V1016I, F1534C and V410L) along with an increase in the mean activity levels of glutathione S-transferases (GSTs), α-esterases, β-esterases and Mixed-Function Oxidases (MFOs) [39,40].

Control of *Ae*. *aegypti* during the attack phase was implemented from week (W) 24 to W27. Traditional vector actions were implemented in both localities (throughout the entire area) and included:

- Removal of potential breeding-habitats of immature mosquitoes through solid waste disposal campaigns (clean up campaigns) with community participation on W25. Activities involved news broadcasting (by different public-media and house-to-house) to inform people the importance of reducing/eliminating disposable breeding sites from their premises by taking them to specific ‘recollection points’ in the locality where MoH staff and municipality dispose them.
- Chemical control of adult *Aedes* (malathion truck-mounted outdoor ULV-fogging) in four weekly applications of both localities (W24-W27) was implemented by MoH personnel following standard procedures (malathion 41.31% EW at dilution ratio 1:2.33, Agroquímica Tridente S.A. de C.V, applied with equipment 1800E-OHV ULV Sprayer, Clarke, at a flow rate of 416 mL/min and moving at 10 km/h allowing an approximate dose of 120 g a.i. of insecticide per ha). ULV-fogging started 1,900 hours and was conducted in a single day application per week per locality; these activities involved house-to-house visits to inform participants about the specific days for the ULV-fogging and people were encouraged to keep their doors-windows open.

### Suppression phase

This phase incorporated mass-releases of *w*AlbB *Ae*. *aegypti* males in SPC (release site) twice a week (Fig 1B and 1D). Below we describe mass-rearing and release protocols. In addition, the municipality conducted a round of ULV spraying with a pyrethroid-based insecticide (Aqua-Reslin Super [0.14% S-Bioallethrin + 10.3% Permethrin + 9.8% PBO]) during W45-W47 (7^th^, 14^th^ and 21^st^ of November) in both study sites, the intervention and control site.

### Establishment and development of a *Wolbachia-*infected *Aedes aegypti* line

*Aedes aegypti* mosquitoes were originally collected as eggs with ovitraps throughout the National Network of Surveillance of the Mexican Ministry of Health [41,42] in 2017 from several neighborhoods of Merida. Eggs were processed according to CENAPRECE [43] and sent to the Department of Microbiology and Molecular Genetics of Michigan State University (MSU), where they were maintained and hatched under standard conditions 80 ± 5% humidity, 26 ± 1°C temperature and photoperiod of L12:D12. At MSU insectary, wild populations of *Ae*. *aegypti* of Merida were backcrossed (7 generations) with *w*AlbB-transinfected *Ae*. *aegypti* WB2 line [10,44]. Eggs from backcross-produced *Ae*. *aegypti* line (hereafter termed *w*AlbB *Ae*. *aegypti*) were returned to the LCB-UADY for their colonization, mass-production standardization processes and preparation of mass production for pilot trials. Supplemental material describes the establishment and development of a *w*AlbB trans-infected *Ae*. *aegypti* from Merida, and other quality control related processes can be also consulted in the supporting information (S2 Appendix).

### Mass-production of *Ae*. *aegypti w*AlbB males for pilot trial

At LCB-UADY (Fig 2A), parental generations were established in preparation for standardization of mass-rearing and quality control processes. The mass-production of male *w*AlbB *Ae*. *aegypti* was based on protocols described previously by Zheng et al. [1] with slight modifications [45–47]. The process includes five steps (Fig 2B): adult rearing, egg hatching, larvae rearing, sex separation, X-ray irradiation and packaging. Quality controls for every step of the mass-production process were established to assure the best quality of the reared mosquitoes infected with *Wolbachia* (*w*AlbB *Ae*. *aegypti*) every reared generation as well as before every mosquito release [28].

The standardized mass-production system for male release consisted in the establishment of 20 adult cages weekly (80 cages monthly) (BugDorm-1Insect Rearing Cages, 30 × 30 × 30 cm) containing 3,000 female pupae and 1,000 male pupae (3:1 ratio of female to male) until adult emergence [46]. Adults were continuously provided with 10% sugar cane solution. Six to seven days-old females were fed with bovine blood during two consecutive days. One day after the first blood-feeding, mosquitoes were provided with oviposition cups to collect eggs every 24 h during two-straight weeks. As part of the quality control process for mass-production, eggs from 2 or 3 oviposition were left in oviposition cups during 48 h for embryonic development and drying under standard conditions at 80 ± 5% humidity and 26 ± 1°C before hatching. After hatching, ≈ 6,500 first instar larvae were added to Wolbaki trays (length × width × height = 58 cm × 38 cm × 4 cm) filled up to 4 L of water at a depth of 1.5 cm [45]. Larvae were fed daily with a mix of 90% fish food (Biofinguerlin) and 10% yeast powder (Pronat Ultra) during six days. When pupae started to develop at day 6, no food was added. On day 7, pupae were collected using a 40-mesh sieve which eliminates residual larvae. Pupae were sex separated by size using the Fay-Morlan method [47,48] with a Mosquito Pupae Sex Sorter (Guangzhou Wolbaki Biotech Co. Ltd). Male pupae collected through mechanical sorter were exposed to irradiation at 45 Gy for 1,000 s to sterilize any residual females using a multifunctional X-ray irradiator (Guangzhou Wolbaki Biotech Co. Ltd). Approximately 65,000 to 75,000 pupae were placed together in a canister (diameter 7.5 cm × height 7.5 cm) with two canisters being simultaneously irradiated.

After irradiation, weekly batches of approximately 1,000 male pupae were transferred into cylindrical containers (14-cm diameter × 24-cm height) with water (0.8-cm depth). Containers with pupae were left 48 h at the package room at 28±1°C until adult emergence. The lid of the container had a hole covered on top with mesh gauze which allows air exchange and prevents the escape of emerging adult mosquitoes. Cotton soaked in 10% solution of sugar cane was placed on top of the gauze as the emergence of adult mosquitoes happens. After 48 h, water was removed through an incision in the wall of the cylinder container by turning upside down. Newly emerged males were left at 27°C overnight in the field lab station for sugar feeding and recovering before being released. Additional quality control measures were used by visually checking to remove any remaining females within adult males. Finally, cylinders with males were then transported to the field for release. Additionally, backup production of 50–100 cylinders (20% extra) with males were prepared each week for field release.

### Mass release of *w*AlbB *Aedes aegypti* males

The release of *w*AlbB *Ae*. *aegypti* males at SPC started on W28 and extended for a total of 24 weeks (6 months) from July to December 2019, covering the rainy season and the period of higher *Ae*. *aegypti* abundance. Approximately 1,270,000 lab-produced *w*AlbB *Ae*. *aegypti* males were released in the 50-ha target area over the 24-week period. Based on our baseline study [25], we estimated releasing 2,000 *w*AlbB *Ae*. *aegypti* males per hectare across 50 ha (total area of SPC) twice a week in two different release days (100,000 male mosquitoes per day, totaling 200,000 male mosquitoes per week). This estimate was based on a target ratio of 10:1 *w*AlbB:Wild Type (WT) recommended for IIT-SIT [1,9,17,18,49–52]. The base number for this 10:1 ratio, following Ritchie et al. [53], was based on a peak density of 168 males per hectare [25] and 8,400 in the 50 hectares of SPC. This release schedule was followed from W28 to W33. Between W34-35, and after seeing a large reduction in total *Ae*. *aegypti* indoors, the MoH reduced the number of males released by 50% (1,000 *Ae*. *aegypti w*AlbB male mosquitoes per hectare twice a week). However, after seeing a “rebound” on indoor adult indices on W37-38, the MoH returned to the original release number (2,000 males/hectare 2/week) until W52 (the end of the suppression phase).

The mass-rearing factory was an approximate 15-minute drive from the release site. Mosquitoes were transported from LCB-UCBE in a vehicle (van) with controlled temperature (22°C) every Tuesday and Friday. Releases occurred in the mornings between 08:00 and 09:30 h. The male mosquitoes were contained in plastic cylinder vases (Ø 14 cm, 25 cm height, 2.8 L), closed with a mesh cap. Cotton soaked with 10% sugar solution was continuously supplied to the males before release. Male releases were conducted by mixed teams (MoH and UADY) every 100 m, in the backyard of houses selected for release and close to mosquito resting areas (e.g., vegetated, shaded, humid areas in the peri domicile or nearby the house) (Fig 1D). After each release, field workers inspected the containers to make sure that all males were released before leaving the study areas.

### Monitoring suppression of wild populations of *Ae*. *aegypti*

As described above and in Che-Mendoza et al. [25], sentinel sampling-stations provided entomological surveillance data within one-hectare areas at both study sites (Fig 1B). *Ae*. *aegypti* populations were monitored during the duration of the study by MoH and UADY staff on a weekly basis using 100 ovitraps/locality (two ovitraps/ha) and 30 BG sentinel traps for outdoor adult collections (one trap per ha) in 24 h-cycles. The material from traps was transported to the Collaborative Unit for Entomological Bioassays of the Autonomous University of Yucatan (UCBE-UADY) for processing. The ovistrips were placed in an incubation chamber (container with a cotton soak with water and covered) for 48 h, then ovistrips were left to dry for 24 h. The hatching of eggs and emergence of adult mosquitoes was carried out under insectary conditions: 26°C ± 2°C, 75% ± 5% RH, L12:D12 (light: darkness). From ovitraps we calculated the mean hatch egg rate, from the number of *Ae*. *aegypti* larvae emerged (36 hours)/total number of eggs per individual ovitrap. The number of eggs and the number of larvae were determined by visual examination using a stereomicroscope. All BG collections were identified to species and sexed. The total and average number of *Ae*. *aegypti* females per trap, in both release and control sites, was determined each week.

To corroborate that the eggs collected belonged to *Ae*. *aegypti*, a weekly sample of 10% of ovistrips were placed for rearing of all larvae until adult emergence. *Aedes aegypti* was initially the only species that lay eggs on ovistrips found at both localities. However, *Ae*. *albopictus* invaded Merida (including suburban areas) [54,55] and we detected its presence in ovitraps at SPC and later in TAH on W37-W38. Consequently, individual examination of ovistrips was needed afterwards. After 72 h hatching period, all larvae were allowed to reach LIII-LIV stage and were identified under a stereomicroscope for their taxonomic determination (to correct the calculation of number of hatched eggs per ovitrap from *Ae*. *aegypti* and *Ae*. *albopictus*).

In addition to traps, six cross-sectional entomological surveys of indoor adult mosquitoes were performed with Prokopack aspirators [37] for a 15 min period per house in a sub-sample of houses with BG traps. *Ae*. *aegypti* is a highly endophilic species that transmits viruses mainly indoors; therefore, for this study we included collections of indoor female *Aedes* using Prokopacks aspirators [37] as a new entomological indicator to assess the impact of combined IIT-SIT. The average number of female *Aedes* per house in both release and control sites was calculated each survey and used as an indicator for the human-mosquito contact reduction in the suppression stage.

### Data management and analysis

The mean number of adult females and egg hatching rates were compared between SPC and TAH sites on six periods: 1) the pre-intervention (W17-W23); 2) the attack phase (W24-W27); the *w*AlbB release phase at 3) 10:1 ratio releases (W28-W33), 4) the adaptive release period (W34-38), and 5) when releases were restored to 10:1 (W39-W44) and finally, 6) when both *w*AlbB (10:1 ratio) and ULV spraying at both sites were conducted (W45-52).

A Generalized Estimating Equation (GEE) analysis was performed to investigate the pre-post effect of the attack phase within each locality, using the log link function and assuming an independent correlation structure. Negative binomial model analyses for female adult collections, and a binary logistic regression analysis for egg hatch rate were applied at each week-period of the suppression phase. Incidence rate ratios (IRR) and Odds Ratios (OR’s) with 95% confidence intervals are presented; and a *p*-value < 0.05 was considered significant. Analyses were performed with STATA 12.0

Entomological indicators were used to calculate a measure of suppression efficacy, as Interventioneff = (1- OR or 1-IRR) x100 [56]. This value, which ranks between 0 and 100, indicates the proportional reduction in *Ae*. *aegypti* in the treatment site, in comparison to the control site.

## Results

**Fig 3:**
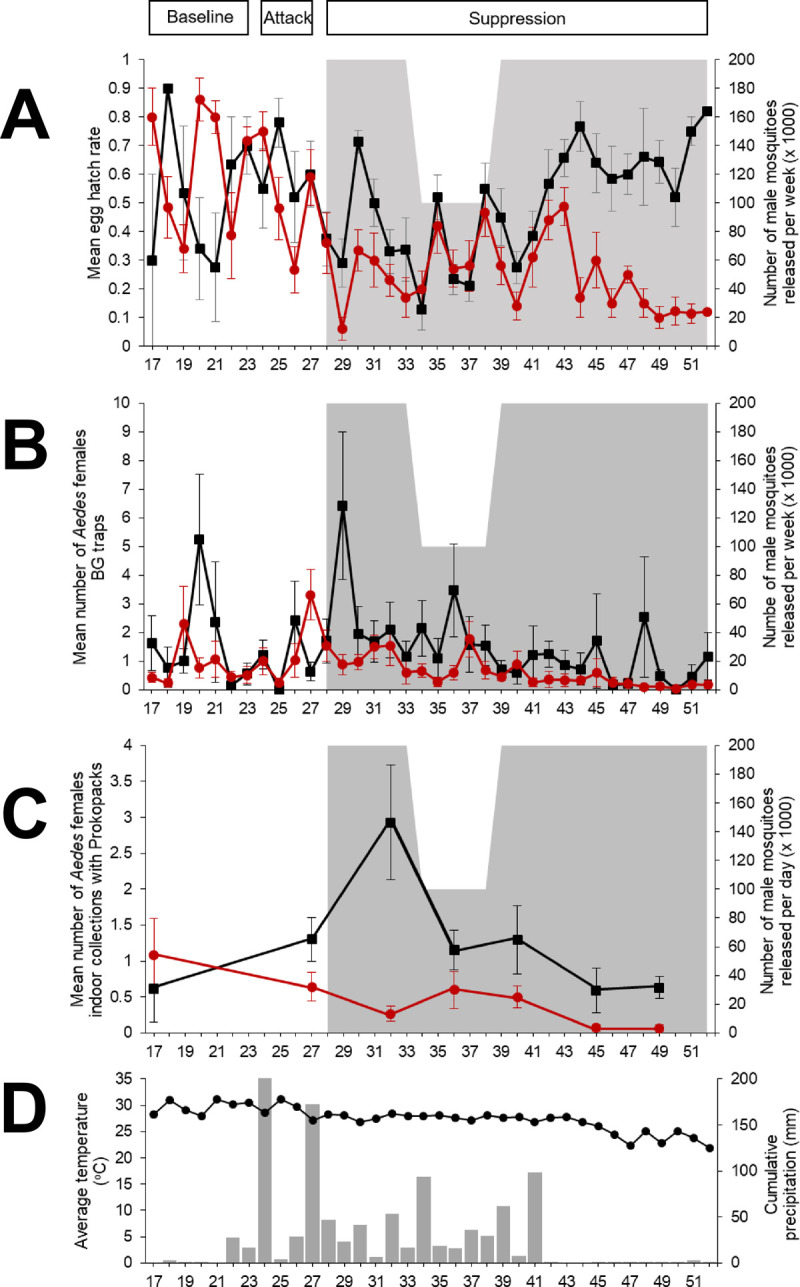
Entomological indicators of population suppression between release (San Pedro Chimay = red line) and control (Tahdzibichén = black line) sites. A) Mean hatching rate (SD) per week, B) Mean No. of females captured with BG traps (SD), C) Mean No. of indoor females captured with Prokopack aspirators, D) Average temperature and precipitation during the study period. Grey-shaded regions indicate release periods. The dashes in the X-axis represent the weeks of the year.

Prior to the intervention, all entomological indices (egg hatch rate, number of females per BG trap and number of females captured with Prokopack aspirators per house) did not differ statistically between SPC and TAH localities (Table 1 and Fig 3A–3D). Mean hatch rate averaged 50% for both localities, whereas adult females per BG averaged 0.9–2.3 per trap and females per house 0.6–1.1 (Table 1 and Fig 3A–3D).

**Table 1:** Analyses of entomological indicators of population suppression between release (San Pedro Chimay) and control (Tahdzibichén) sites. Incidence rate ratios (IRR) and odds ratios (OR) with 95% confidence intervals are shown. Intervention efficacy ([1- OR or 1-IRR] x100) is shown.

Egg hatch rate	Period	Study site	OR	95% C.I.	P value	Intervention efficacy
Baseline	W17-20	Release	7.67	0.7–83.73	0.095	NA
		Control				
	W21-23	Release	1.24	0.17–9.25	0.835	NA
		Control				
Attack phase	W24-27	Release	1.25	0.25–6.23	0.785	—
		Control				
Suppression phase	W28-33	Release	0.23	0.06–0.9	0.034*	**76.5%**
		Control				
	W34-38	Release	1.03	0.35–3.0	0.961	—
		Control				
	W39-44	Release	0.12	0.01–0.98	0.048*	**88.0%**
		Control				
	W45-52	Release	0.08	0.01–0.66	0.019*	**91.9%**
		Control				
**No. outdoor females/ BG trap**	**Period**	**Study site**	**IRR**	**95% C.I.**	**P value**	**Intervention efficacy**
Baseline	W17-20	Release	0.42	0.15–1.12	0.082	NA
		Control				
	W21-23	Release	0.68	0.19–2.39	0.543	NA
		Control				
Attack phase	W24-27	Release	1.06	0.43–2.59	0.903	—
		Control				
Suppression phase	W28-33	Release	0.45	0.26–0.79	0.005*	**54.7%**
		Control				
	W34-38	Release	0.39	0.2–0.75	0.005*	**61.4%**
		Control				
	W39-44	Release	0.50	0.24–1.03	0.060	50.0%
		Control				
	W45-52	Release	0.25	0.09–0.68	0.007*	**75.2%**
		Control				
**No indoor females per house**	**Period**	**Study site**	**IRR**	**95% C.I.**	**P value**	**Intervention efficacy**
Baseline	April (W17)	Release	1.77	0.35–9.02	0.494	NA
		Control				
Attack phase	July (W27)	Release	0.49	0.21–1.16	0.102	—
		Control				
Suppression phase	August (W32)	Release	0.09	0.04–0.23	<0.001*	**90.9%**
		Control				
	September (W36)	Release	0.52	0.22–1.25	0.146	47.7%
		Control				
	October (W40)	Release	0.39	0.15–0.98	0.045*	**61.4%**
		Control				
	November (W45)	Release	0.12	0.02–0.71	0.02*	**88.4%**
		Control				
	December (W49)	Release	0.11	0.02–0.46	0.003*	**89.4%**
		Control				

The attack phase included routine vector control measures to lower, at both localities, mosquito populations prior to the mass-release of *w*AlbB *Ae*. *aegypti*. One round of removal of disposable and potential breeding-habitats from SPC and TAH, and application of malathion once a week per 4 weeks during the attack phase, did not lead to a measurable reduction in entomological indicators compared to baseline (GEE, hatch rate: Coef = 0.32, P = 0.63 for SPC, Coef = 0.13, P = 0.86 for TAH; BG female collections: Coef = 0.03, P = 0.56 for SPC, Coef = 0.003, P = 0.92 for TAH; indoor female collections: Coef = -0.12, P = 0.60 for SPC, Coef = 0.23, P = 0.31 for TAH). Furthermore, no statistical difference was observed between intervention and control localities during the attack phase for hatch rate, outdoor females collected by BG traps or indoor females collected with Prokopacks (Table 1).

Throughout the suppression phase, we observed an overall significant reduction in entomological indicators in SPC compared to TAH (Table 1 and Fig 3A–3D). Egg hatch rates significantly decreased by 76.5% (OR = 0.23, 95% CI = 0.06–0.9) compared to the control locality during the first phase of releases (W28-33) when the initial number of males released per week (200,000) was implemented. A lack of significance in egg hatch rate between localities was quantified during the period of adaptive release (W34-38), whereas a significant 88% (OR = 0.12; 95%CI = 0.01–0.98) to 92% (OR = 0.08; 95% CI = 0.01–0.66) reduction was observed during the remaining release period (W39-52) (Table 1 and Fig 3A–3D). As part of the project’s enhanced entomological surveillance, we identified the *Aedes* spp. laying eggs in ovistrips. For SPC, 100% of emerged adults were *Ae*. *aegypti* from early 2019 to W38. Of note, starting at W39-, we detected a variable presence of *Ae*. *albopictus* in both localities, with an overall average of 22% hatched larvae between W39 and W51 (Table E in S2 Appendix).

In general, the number of *Ae*. *aegypti* females collected outdoors using BG traps were also significantly lower in the release compared to the control locality during the suppression phase (Table 1 and Fig 3A–3D). For the first phase of releases (200,000 male mosquitoes per week, W28-W33) models quantified a significant reduction in the number of outdoor females of 54.7% (IRR = 0.45; 95% CI = 0.26–0.79) compared to the control locality. Such a significant reduction increased to 61.4% (IRR = 0.39; 95%CI = 0.2–0.75) during the period of adaptive releases (W34-38). The reduced release rate had a delayed effect on outdoor female abundance, evidenced by the lack of statistical significance in the number of outdoor females between release and control localities on W39-44 (IRR = 0.50; 95% CI = 0.24–1.03; P = 0.06). During the last weeks (W45-W52), after restoring the release rate to 200,000 male mosquitoes per week, the model estimated a significant reduction of 75.2% (IRR = 0.25; 95% CI = 0.09–0.68) in the release locality compared to the control.

Overall, the largest effect of the intervention during the suppression phase was quantified for the number of indoor *Ae*. *aegypti* females per house (Prokopack collections) (Table 1 and Fig 3C). A month after the start of releasing 200,000 male mosquitoes per week (August W32), the number of indoor *Ae*. *aegypti* females were 90.9% lower (IRR = 0.09, 95% CI = 0.04–0.23) in SPC compared to TAH. In September (W36), when adaptive releases were conducted, the number of indoor *Ae*. *aegypti* females did not differ significantly between localities (IRR = 0.52, 95% CI = 0.22–1.25, p = 0.146). The efficacy of the intervention in reducing *Ae*. *aegypti* female abundance increased significantly compared to the TAH after W36 (Table 1). On W40 (October), efficacy was 61.4% (IRR = 0.39; 95% CI = 0.15–0.98) (Table 1). November (W45) sampling led to an efficacy of 88.4% (IRR = 0.12, 95% CI = 0.02–0.71) and December (W49) sampling to reductions of 89.4% (IRR = 0.11, 95% CI = 0.02–0.46) in the number of indoor *Ae*. *aegypti* females.

## Discussion

We confirm in an *Ae*. *aegypti* endemic locality of Mexico that males produced with the IIT-SIT approach and using the *w*AlbB strain can reduce natural populations of *Ae*. *aegypti* females area-wide. We also demonstrate that *Wolbachia*-infected *Ae*. *aegypti* males can be mass-produced and mass-released successfully and implemented as part of an IVM plan with the leadership of the MoH in Mexico. As reported in the quality control studies (S2 Appendix), the processes and mosquito rearing and handling conditions at the LCB-UADY preserved *Wolbachia* infection between generations with high rates of CI and produced competitive males, all appropriate traits for the sustainability of IIT-SIT for *Ae*. *aegypti* suppression [9].

Our pilot study adds to the growing body of evidence about the entomological impact of *Wolbachia*-based biocontrol to suppress *Ae*. *aegypti* populations [3–6,17,18,57,58]. Available examples of this IIT approach include (mostly) demonstrations in USA [5,18] and Australia [6] showing significant (>70%-95%) seasonal suppression of *Ae*. *aegypti* female density. A modification of IIT, whereby pupae are irradiated to sterilize any females that are unintentionally released with the males (IIT-SIT), has shown similar levels of impact [3,17,58]. In Thailand, Kittayapong et al. [17] demonstrated in a pilot study that IIT-SIT reduced *Ae*. *aegypti* females’ density per household by 97.3%. The most extended experience using IIT-SIT as part of a governmental vector control program is from Singapore, where its efficacy to control local *Ae*. *aegypti* populations have shown different levels of suppression (from 50%—>90%) after different phases from 2016-to date [3,58]. Our results from Yucatan confirm that *w*AlbB infected *Ae*. *aegypti* males produced with the IIT-SIT method reduced the natural density of *Ae*. *aegypti* density and egg hatch rate by 50–90% depending on the entomological index, the study phase and rate of male releases.

Overall, results from our study show comparable entomological endpoints (egg hatching, outdoor-adult females collected with BG traps and indoor female collections) and comparable efficacy as those reported from previous experiences in Thailand and Singapore [3,17,58]. Egg hatching rate is used as a correlate of CI in field evaluations [1,3,17,27,58]. This method is only a valid replacement of conducting mating when treatment and control areas can be compared (as our study). In Yucatan, egg hatch rate was strongly associated and instantly impacted by the ratio of *w*AlbB:WT mosquitoes released, showing a rapid drop after initial (inundative) releases, then an increase when release rates were halved, to then trend to a >90% reduction once releases were increased back to 200,000 male mosquitoes per week. Therefore, hatch rate could be considered more an index of operational efficacy than of entomological impact and, in our study, could help the MoH inform whether they must modify their release rates to achieve a desired level of efficacy. The reduction of egg hatching success -and increased egg mortality- observed here, induced by CI in addition to natural factors, mainly high or low temperature, low humidity, and desiccation conditions as well as senescence, predators, and parasites, is expected to have strongly influenced the population persistence [59] at the release site in comparison to TAH site.

The main entomological endpoint of a *Wolbachia*-based approach for population suppression is the reduction of natural vector populations, particularly female mosquitoes. Our research provides further evidence that an IIT-SIT approach, preceded by an attack phase focused on source reduction and outdoor space spraying, can significantly reduce the abundance of outdoor and indoor female *Ae*. *aegypti* particularly during the peak-period of population abundance. Indoor female *Ae*. *aegypti* density is considered one of the closest entomological measures of arbovirus transmission risk [60,61]. Indoor female *Ae*. *aegypti* was the index most significantly impacted by *w*AlbB releases, showing an impressive 80–90% reduction throughout the study and when releasing 200,000 male mosquitoes per week. Our findings of impact on indoor female density reductions, albeit entomological, provide important evidence supporting the potential of IIT-SIT in reducing the risk of *Aedes*-borne virus transmission.

Adult-based entomological indicators were sensitive to the male release ratios, but delayed 1–2 weeks, showing that when adaptive ratios are implemented following an underestimated 5:1 ratio (100,000 male mosquitoes per week), suppression cannot be sustained. Our findings point to the complexity of real-time adult surveillance and further studies will be needed to prove if “adaptive” release rates or “adjusted” number of males/area/time can be operationally effective. We envisage that under operational activities of the MoH, rigorous, systematic, and high-quality entomological monitoring will not be feasible or practical, leading adaptive approaches more as a research approach than a public health implementation of IIT-SIT.

A unique component of this project has been the integration of *Wolbachia* population suppression within the IVM plan of Yucatan, rather than as a stand-alone intervention. This integration not only allowed better implementation in the field, by engaging field technicians in releases, but also a closer connection of the intervention with the community. In consensus with the MoH, it was considered logistically complementary to include routine activities before *Wolbachia* releases. We show that such interventions-maintained *Ae*. *aegypti* indices as they were in the baseline period of low abundance and achieved the intention to maintain the 10:1 overflooding ratio compared to maximum baseline density. A 10:1 rate (or higher) seems to have worked in various studies aiming for population suppression of *Ae*. *aegypti* [3,4,9]. In our experience, it seems that a single application rate (e.g., number of males/area/time) of 10:1 was more adequate to suppress mosquito populations than a 5:1 ratio.

Any country considering the implementation of strategies with releases of incompatible/sterile-male *Aedes* mosquitoes will have to augment the capabilities of its entomological surveillance system [62]. Ovitraps represent an economic and simple method for monitoring *Aedes* mosquito populations which is already in use by the Mexican MoH [31,41], and an established infrastructure to monitor egg hatch rates after *Wolbachia* release. Adult collections are not systematically done as part of routine activities, although “entomo-virological surveillance” (adult collections and arbovirus detection) is currently being conducted with portable aspirators such as Prokopack aspirators in a few Mexican States to determine the prevalence and abundance of female mosquitoes and infection with DENV, CHIKV and now ZIKV [61,63–65]. Therefore, indoor adult female collections, which represent the epidemiologically important target [17,18,33,66], can be more feasible to implement than outdoors collections (with BG traps or others).

The unexpected finding of *Ae*. *albopictus* in the study sites was a challenge for intervention monitoring using ovitraps since their eggs are morphologically indistinguishable from those of *Ae*. *aegypti*. We modified our protocols to revise ovistrips individually, hatching eggs and separating larva which was labor-consuming and occupied a large amount of time, personnel, and space. Species separation at the adult stage from BG and Prokopacks was less complicated, but still, it was an unforeseen task. Studies from our group show that *Ae*. *albopictus* is invading the Yucatan State and is more commonly found in urban areas coexisting with *Ae*. *aegypti* [54,55], which complicates species-specific methods of vector control such as IIT-SIT, SIT or *Wolbachia*-based population replacement. Here, after several weeks of intervention, we found that *Ae*. *albopictus* larvae hatched from field-collected ovistrips which suggests a potential niche shifting can occur by the replacement of *Ae*. *aegypti* by *Ae*. *albopictus* in the intervention areas. However, currently there is no robust published data suggesting that *Wolbachia*-based control interventions may result in mosquito populations replacement of niche space between species. Additional follow-up-controlled studies will be critical to better understand whether this phenomenon may occur in intervention areas using *Wolbachia*-infected mosquitoes.

Furthermore, the invasion of *Ae*. *albopictus* will influence IIT-SIT perceived efficacy by the community, as they may not experience reductions in mosquito bites due to the replacement of *Ae*. *aegypti* by *Ae*. *albopictus*. During the project we tried to expand the knowledge about the different species of mosquitoes, and the male-female characteristics of local and released mosquitoes in the field through community education, workshops, and household-visits before/after the releasing activities. A complex scenario like this provides further justification for the integration of IIT-SIT within an IVM plan, as insecticide-based approaches (outdoor ULV) can impact the more peridomestic *Ae*. *albopictus* while IIT-SIT impacts indoor *Ae*. *aegypti*. Another important unforeseen aspect of *Ae*. *albopictus* invasion is that its presence can boost IIT-SIT impact. It has been documented that, *Ae*. *albopictus* satirizes *Ae*. *aegypti* in some settings, leading to inviable mating and population displacements [67]. The presence of *Ae*. *albopictus* could, in the future, add another source of sterility to *Ae*. *aegypti*, potentially displacing it from local areas where both species interact. Finally, as *w*Pip transfected *Ae*. *albopictus* also are used for population suppression [1], one can also envision a program that releases males from both species to catalyze overall reductions in human-mosquito contacts.

This pilot-study was only able to implement only one suppression phase [9]. It’s no surprise that many demonstration studies only run for one season/year since most funding agencies/grants subsidize projects/budgets for short periods of time (1–2 years). We developed, with support from Mexico’s Science Council (CONACYT) and U.S. Agency for International Development (USAID), a 3-year project which included two successfully accomplished milestones: the establishment of a facility with a mass rearing system (in operation) and a pilot study [25]. Coordinating the logistics of a male release project from zero is not trivial [9]. For example, preparation for a study in a new area includes ca. 1-year before actual mass field-releases take place [62]. We intended to continue with our IVM at least for a second year, but funding limitations and the COVID-19 contingency impeded its extension.

Dobson [9] recently described various aspects that influence the economics of male release programs. Maintenance of a population to low levels (i.e., after successful suppression) can be much cheaper than the first year of suppressing an established population because of biological aspects (reduced natural egg banks, known seasonality and timing for releases), logistics, social and regulatory agencies permission, etc. In addition, it is recommended that the most cost-effective approach will be an integration of two or more control methods [3,9]. Specifically, traditional pesticides can be used to reduce high densities of target populations, followed by male releases to sustain low mosquito densities, or eliminate the population. Chemical adulticides that kill adult males also may be integrated with male releases, but the applications of chemicals and males should be coordinated to take place at different times. This IVM integration of *Wolbachia* will be more logical for population suppression approaches than for replacement, as the latter depends on mosquito dispersal and population growth for its establishment. The contrasts between both approaches are not minor and could lead to different choices by MoHs depending on their operational structure and epidemiological conditions.

Subject to a future analysis and report describing the costs of producing, releasing, monitoring, and scaling interventions releasing mosquitoes within MoH institutional programs, this project allowed generating basic information on total direct costs of IIT-SIT and its comparison with the standard application of truck-mounted ULV, both conducted by the MoH. The cost estimates were based on actual expenses in products, personnel, petrol for vehicles/equipment, and consumables etc. identified for an area of 50 ha *v*.*gr*. like San Pedro Chimay (Table 2). The perspective of this exercise is from the viewpoint of the funder/user, in this case the government, and thus only direct costs are included.

**Table 2:** Cost estimates table (USD) for the implementation of ULV spraying and release of IIT-SIT *w*AlbB males identified for an area of 50 ha *v*.*gr*. San Pedro Chimay.

**ULV spraying** One application of truck-mounted outdoor ULV-fogging of malathion covering blocks of the whole locality	**Costs of “product”**	**Description**	**Cost per-50 hectares**
	Insecticide	Malathion 41.31% EW at dilution ratio 1:2.33, applied with equipment 1800E-OHV ULV Sprayer, Clarke, at a flow rate of 416 ml/min and moving at 10 km/h allowing an approximate dose of 120 g A.I. of insecticide per ha.	825.0
	**Costs of Implementation**	**Description**	**Cost per-50 hectares**
	Personnel (Salaries)	2 technicians with a salary of 400 USD per month. Calculation is made for 4 h/labor (with a vehicle they can cover 50 hectares in approx. 2 h, plus 2 hours for preparing the mix and calibration of the equipment).	13.33
	Petrol (vehicle)	An 8-cylinder pickup truck consumes an average of 15 liters per day (60 hectares). Price per liter is 20.86 pesos. We considered the cost of 13 liters for 50 hectares	13.56
	Petrol for the ULV-equipment	Considering the consumption of 10 liters for 50 ha.	10.43
	Stationary/office materials		1.50
	**Total**		**863.82**
**IIT-SIT** Mass-releases of *w*AlbB *Ae*. *aegypti* males twice a week	**Costs of “product”**	**Description**	**Cost per-50 hectares**
	IIT-SIT *w*AlbB *Ae*. *aegypti* males produced at LCB-UADY	Release of 4,000 *w*AlbB *Ae*. *aegypti* males per hectare (2,000 males released per hectare twice per week).	340.0
	**Costs of Implementation (Release)**	**Description**	**Cost per-50 hectares**
	Personnel (Salaries)	2 technicians with a salary of 400 USD per month per person. Calculation is made for 4 hrs./labor (with a vehicle they can cover 50 hectares in approx. 4 hrs.) and working two days (two releasing days)	26.65
	Petrol (vehicle)	An 8-cylinder van consumes an average of 15 liters per day (60 hectares). Price per liter is 1.043 USD. We considered the cost of 13 liters for 50 hectares	27.12
	Release items/consumables	Includes releasing vases and boxes for storage and transportation.	8.5
	Stationary/office materials		1.50
	**Total**		**403.77**

The product (commercial insecticide e.g Malathion) needed for one application covering blocks of the whole locality (50 ha) delivered as ULV spraying costs 825 USD/week. The delivery of insecticide in the field, this is, its operational application by staff of the MoH in Yucatan costs 38.82 USD. Therefore, the total cost of one application of ULV spraying with Malathion for a 50-ha area is 863.82 USD/week.

We found that the cost of producing IIT-SIT *w*AlbB males is competitive, provided a building for the mosquito factory is already in place (i.e., no construction costs are included). The LCB-UADY committed to this project a production of 4,000 *Ae*. *aegypti* males to be released in a 50 ha-area every week (2,000 males released per hectare twice per week), with corresponding costs of 340 USD. Implementation costs (field personnel for releases, petrol for vehicles, and other consumables) was 63.77 USD, leading to a total cost of releasing 4,000 *Ae*. *aegypti* males/50 ha to be 403.77 USD per week (two releases per week).

We have not included the cost of media publicizing/community sensitization and engagement or entomological surveillance/monitoring; but a large difference of the costs of implementation between ULV-spraying vs. “rear and release” of *Ae*. *aegypti* can be expected. Methods currently employed by the MoH such as ULV spraying of insecticides are well known and do not require further explanation to the population, beyond the details that are regularly provided in vector control campaigns. In contrast, we identified that IIT-SIT would require intense and long-term social/community work and the delivery of specific messages to characterize and describe the processes related with mass-releases of male mosquitoes as an IVM strategy for vector control. Such costs may be reduced over time, as the intervention is rolled-out to more places and communities grow awareness of it. In the case of entomological surveillance/monitoring, most programs (if any) only do sporadic pre-post intervention surveillance activities (particularly if temporary methods such as ULV-praying are applied) and do not carry out systematic surveillance with ovitraps or adult collections. Proper entomological surveillance should not be exclusive to innovative methods such as IIT-SIT but should be common to all interventions.

While ample mention of the “sustainability” of *Wolbachia* methods for population suppression or population replacement exists [8,9], there are no well-defined examples of cost estimates of this approach in comparison with existing practice. Building sustainable control programs, even with the inclusion of innovative technologies, in the face of technical, operational, and financial limitations is still a challenge and will require the engagement and collaboration of local communities. Novel approaches for vector control should be adapted to the capacities of the MoH and governments (who will develop and finance the actions), as well as the socioeconomic and environmental context and achieve short- and longer-term objectives for human well-being.

The terms suppression and reduction define theoretical endpoints that are poorly defined for SIT, IIT and combined IIT-SIT. To date, as far as we are aware, there is no evidence of a “fully successful” population suppression case for *Ae*. *aegypti* (although the recent Australian study reported over 95% *Ae*. *aegypti* suppressions in the following season after releases in one of the 3 treatment landscapes) [6]. Indeed, our study and all studies referenced above have demonstrated significant reductions of important entomological indicators but no local elimination of *Ae*. *aegypti*. Population reduction approaches aiming for “complete suppression” will require continuous releases of males over a long time-period and approaches to mitigate the migration of mosquitoes from surrounding (untreated) areas [8]. The proof-of-concept here reported a combined IIT-SIT approach in reducing natural populations of *Ae*. *aegypti* should be useful as evidence for an alternative or complementary approach to be applied in several countries. As stated by the Vector Control Advisory Group (VCAG) of WHO, this combined IIT-SIT technology has the potential for long-term control of *Ae*. *aegypti* mosquitoes [68].

With regards to the scalability of our findings into larger cities, it is undeniable that such expansion will be challenging. Our team is uniquely positioned to make that transition into the city of Merida because: a) our facilities were designed to operate at the scale of production required for larger areas; b) our pilot study taught valuable lessons about community acceptance and release regimes; c) by involving the MoH we ensured that the strategy is integrated with other approaches currently in use to control *Ae*. *aegypti*. Particularly, we are currently considering and designing interventions for vectors, and importantly on disease prevention, that are targeted interventions in high-risk areas (hot-spots) within endemic localities [69]. Control of zones within urban areas of Singapore recently demonstrated that releases of males produced with IIT-SIT can dramatically reduce wild type *Ae*. *aegypti* populations and dengue incidence in the targeted areas [3].

## Supporting information

S1 Appendix Community engagement. Table A: Social studies. A community-lead approach was developed, divided into four phases, each one with key milestones and activities described in the following table.
(DOCX)

S2 Appendix Quality control processes of the mass-production of *Ae*. *aegypti*. Table A: Female contamination rate during five months for pilot trial. Table B: Cytoplasmic incompatibility (CI) from the different mating crosses. Table C: Irradiation treatments on male and female *Wolbachia*-infected *Ae*. *aegypti*. Table D: Competitiveness between *Wolbachia*-infected males (*w*AlbB *Ae*. *aegypti*) and unirradiated wild type males (MID). Mean (± SD) are shown. Table E: Percentage of *Ae*. *albopictus* larvae hatched per individual ovistrip (No. *Ae*. *albopictus* larvae/Total *Aedes* eggs x 100) at San Pedro Chimay (SPC) and Tahdzibichén (TAH).
(DOCX)

S1 Fig Anal segments of *Ae*. *aegypti* pupae, pupae size and detection of *Wolbachia*. (A, C) ventral and lateral (B, D) views of pupal paddles, showing dimorphism characters between males (A, B) and females (C, D). Red arrows indicate the location and morphology of the genital lobes in male and female *Ae*. *aegypti* mosquitoes. Abbreviations: P, paddle; PS, paddle seta; LS, lateral seta. Pupae sizes of male and female *Ae*. *aegypti* mosquitoes. (E) Representative images of male and female pupae (Magnification: 50X). Ventral view of female (left) and male (right) pupae. Red lines show the diameter (mm) of the cephalothorax in the ventral position. (F) Pupae size diameter (mm) of female (n = 125) and male (n = 125) mosquitoes (F11, -13, -16, -18). Data represents the media plus-minus the standard error. (G) Detection of *Wolbachia* genome in male *Ae*. *aegypti* mosquitoes reared under laboratory conditions. Total genomic DNA was extracted from two different generations (F19, n = 29, F30, n = 29) of laboratory-reared adult male *Ae*. *aegypti* mosquitoes artificially infected with *Wolbachia* stain B. Representative image of *Wolbachia* infection detected in male mosquitoes obtained from generations F19 (n = 11) and F20 (n = 11). Genomic DNA from a female *Ae*. *aegypti* (F6) artificially infected with *Wolbachia* strain B (*w*AlbB *Ae*. *aegypti*) (lane 12, top image), and a *Wolbachia* free native *Ae*. *aegypti* from Yucatan (lane 12, lower image) were used as positive and negative controls of the assay, respectively. DNA marker (100 bp). Agarose gel (1%) stained with SYBR safe. PCR positive amplicon: ~600 bp.
(TIF)
