# Supplementary material for: Pilot trial using mass field-releases of sterile males produced with the incompatible and sterile insect techniques as part of integrated Aedes aegypti control in Mexico
Source: PLoS Negl Trop Dis. 2022 Apr 26;16(4):e0010324. doi: 10.1371/journal.pntd.0010324 (PMC9041844; doi:10.1371/journal.pntd.0010324)
Supplement: S1 Appendix — Table A: Social studies. A community-lead approach was developed, divided into four phases, each one with key milestones and activities described in the following table. (DOCX) [file pntd.0010324.s001.docx]

Supporting information

# S1 Appendix. Community engagement

# Table A. Social studies. A community-lead approach was developed, divided into four phases, each one with key milestones and activities described in the following table.

| **Phase** | **Milestones** | **Activities** |
| --- | --- | --- |
| Phase I. Preparation of the community | i) Identification, contact and building bounds of confidence with community leaders and key stakeholders.  ii) Communication of the goals of the project within the community through house-to-house visits and culturally appropriate materials *v.gr.* brochures, posters, promotional-video, and educational documents. | Organization of meetings a year prior to the implementation of the vector control activities (2018) with municipal authorities and a local committee of representatives of the community. Presentation of the objectives, expected outcomes, and developed together a framework of collaboration and agreements.  From this phase and forward, we arranged monthly meetings with local leaders (usually on Mondays) to present and discuss the progress of the activities, including both the positive results but also the barriers and challenges identified for the implementation of the project. The project established a mechanism of social communication (via the project team) to answer questions or resolve concerns reported by the residents of the study site.  After the engagement with local leaders and in collaboration with the MoH, we performed house-to-house visits (N=305) inviting the whole community to participate in the project. Since the beginning, we identified the project needed to design specific messages to characterize and describe the processes related with mass-releases of male mosquitoes as an IVM strategy for vector control, which were done through demonstration activities in workshops and home visits.  Cultural identity of the project was an important factor for the communication with the population. Mayan identity is very important in Yucatan. The phrase "Uts koxol” (“good mosquitoes” in Mayan language) was used by the project. |
| Phase II. Pre-releasing community activities | i) Identification of the strengths and cultural barriers for the implementation of the project through social studies and workshops with key leaders of the community.  ii) Enrollment and acceptance of the families willing to participate during all the activities of the project thought house-to-house visits and cultural-sensitive materials such as brochures, posters, promotional-videos, and educational documents. | Quantitative surveys and qualitative interviews were performed to characterize the local context of the community, demographics data, knowledge, and social experience on mosquito-borne diseases, emphasizing the perception towards traditional and new methods to control *Aedes*-vector in the locality.  One of the major concerns was to understand the perception of the community and the barriers for mass-releases of male mosquitoes. Such a new technology must be well understood for its successful introduction and sustained control. |
| Phase III. Releasing activities in the whole site | i) Engagement of participants from the community through educational workshops reinforcing goals, commitments, and benefits of the intervention.  ii) Reinforcement of the social license of the leaders and the community for the releasing-mosquito process and design a collaborative plan for this activity. | An educational intervention based on PRECED-PROCEEDE model for community adoption of *Ae. aegypti* control with male mosquito releases was developed. Eleven demonstrative workshops were organized at local government facilities to increase the awareness to prevent mosquito-borne diseases, participatory talks about the life cycle of *Ae. aegypti* and its diseases transmitted to humans, and demonstrative explanations of the intervention, the benefits, and the commitments of the participants.  The authorities from the release site gave their full support to perform the study. They played an important role as communicators of the project and active monitors of positive and negative impacts produced.  In agreement and consent of householders, we selected strategic releasing spots (one per block through the whole community). Families that accepted to become part of the intervention (release points), were in-depth informed about the releasing-schedule and provided with extra educational material to reinforce the knowledge of the intervention, and they became local promoters of the benefits and achievements of the project. |
| Phase IV. Post-releasing follow-up activities | i) Follow-up/educational activities to reinforce goals, commitments and benefits of the intervention such as house-to-house visits, community meetings, scientific tours to the “mosquito factory”.  ii) Anthropological assessment of the perceived benefits of the project and recommendations for further scaling-up initiatives. | A set of participatory workshops and educative activities were performed at schools addressing the same topics but adapted to scholar populations: 1 puppet theater at the kinder garden, 6 educative workshops at the elementary and 5 educative workshops and mosquito-releasing demonstrations at the secondary levels. In addition, a scientific tour to the “mosquito factory” was organized where students and professors learnt more about the whole mosquito mass production processes.  An anthropological assessment was conducted to address the benefits perceived by the community and its local leaders about the IVM project that include traditional and innovative strategies such as the implementation of biological control of *Ae. aegypti* with *Wolbachia*.  Finally, press releases were published by local and international newspapers promoting the project as an important Integrated Vector Management to reduce mosquito population in Yucatan, Mexico. |
