# Supplementary material for: Pilot trial using mass field-releases of sterile males produced with the incompatible and sterile insect techniques as part of integrated Aedes aegypti control in Mexico
Source: PLoS Negl Trop Dis. 2022 Apr 26;16(4):e0010324. doi: 10.1371/journal.pntd.0010324 (PMC9041844; doi:10.1371/journal.pntd.0010324)
Supplement: S2 Appendix — Table A: Female contamination rate during five months for pilot trial. Table B: Cytoplasmic incompatibility (CI) from the different mating crosses. Table C: Irradiation treatments on male and female Wolbachia-infected Ae. aegypti. Table D: Competitiveness between Wolbachia-infected males (wAlbB Ae. aegypti) and unirradiated wild type males (MID). Mean (± SD) are shown. Table E: Percentage of Ae. albopictus larvae hatched per individual ovistrip (No. Ae. albopictus larvae/Total Aedes eggs x 100) at San Pedro Chimay (SPC) and Tahdzibichén (TAH). (DOCX) [file pntd.0010324.s002.docx]

**S2 Appendix.** **Quality control processes of the mass-production of *Ae. aegypti***

# Female contamination rate

The female contamination rate (FCR) is a key step in quality control for mass-production. The FCR was monitored at both pupal and adult stages weekly during the pilot trial (August – November). Before irradiation, each batch of sex-sorted pupae was checked randomly, selecting three groups of approximately 1,000 pupae that were sex-determinate under microscopic examination of their terminalia. The batch of pupae sampled qualified for release if the FCR was below 1%. If the FCR was over 1%, mechanical sex separation and manual screening of the batch of pupae was repeated until the FCR was less than 1% [1].

SIT and IIT methods required the release of massive numbers of sterilized/incompatible male mosquitoes acting as mating competitors with fertile wild males [2-6]. Hence, it is important to ensure a homogeneous dispersal of only male *Ae. aegypti* mosquitoes while maintaining their quality, leading to a homogeneous *Wolbachia* infected-to-wild male ratio. Based on the morphology dimorphism of the genital lobes right below pupal paddles (S1A-D Fig), we differentiated between male and female pupae of *Ae. aegypti* found a female contamination ratio (FCR) 0.02% (±0.02) (Table A). In conclusion, at least 99 % of incompatible males were purely separated from females, which demonstrated high efficiency in the manual sex pupae separation process during this pilot intervention and quality control process of the mass-production.

# Table A. Female contamination rate during five months for pilot trial.

| **Month** | **Lot number/week**  **3,000 pupae** | **Male** | **Female** | **FCR (±SD)** |
| --- | --- | --- | --- | --- |
| July | 12 | 12,175 | 2 | 0.0161 (0.0004) |
| August | 13 | 12,837 | 2 | 0.0146 (0.0004) |
| September | 12 | 12,780 | 2 | 0.0149 (0.0003) |
| October | 12 | 12,621 | 2 | 0.0168 (0.0004) |
| November | 13 | 12,916 | 2 | 0.0162 (0.0004) |

# Pupal size

Pupal size measurement was done weekly for mass-production during pilot trial. Also, every three months, this process is performed as an internal quality control process of the mass-rearing system. A sample of 30 pupae-derived females and 30 pupae-derived males was randomly selected and placed on a glass slide and measured as previously described by Timmermann and Briegel [7]. Briefly, dried pupae were placed on a heating plate at 55 ºC for 3 min for immobilization. The width of cephalothorax was measured with their ventral view upward using a digital Dino-Lite® and DinoCapture 2.0 software version 1.5.30.A.

In addition to the morphology dimorphism observed on their genital lobes, pupae sizes are also considered an important feature for sexing *Ae. aegypti* mosquitoes for mass rearing under laboratory conditions. Here, we found that the cephalothorax size average of male pupae was 1.00 mm (± 0.03) and 1.21 mm (± 0.05) for females (S1E-F Fig).

# *Wolbachia* infection in male *Aedes aegypti* mosquitoes: Assuring *Wolbachia*infection per mosquito generation and before field-release.

The successful establishment of the *Wolbachia* suppression campaign primarily relies on the good selection of suitable amounts of *Wolbachia* infected male mosquitoes reared under laboratory conditions. Here, as part of the quality control for mass releasing of male *Wolbachia*-infected *Aedes aegypti*, we optimized a PCR assay that detects the presence of *Wolbachia*infection every generation of adult mosquitoes reared under laboratory conditions as well as before every release campaign. To do so, a total genomic DNA extraction was performed using a DNeasy kit from blood and tissue (Qiagen, Hilden, Germany), following the manufacturer's instructions. The presence of *Wolbachia* DNA genome (*w*Alb+) in male mosquitoes is determined in a total of 29 individual adult males *Ae. aegypti* mosquitoes per every laboratory-reared generation using PCR with *Wolbachia* B specific primers *wsp* 81F (5′-TGGTCCAATAAGTGATGAAGAAAC-3′) and *wsp* 691R (5’-AAAAATTAAACGCTACTCCA-3’) which amplify a fragment (~ 600 bp) of the *Wolbachia* surface protein gene (*wsp*). An endpoint PCR protocol was performed using a Mastercycler EP Gradient-Thermal-Cycler (Eppendorf) and a Taq DNA Polymerase kit and amplification parameters previously established [8]. Here, we show a representative image of the detection of *Wolbachia* genome in two sequential mosquito generations (e.g. F19 and F20) as determined by the amplification of a ~600 base pairs product using PCR (S1G Fig). Nevertheless, the infection with *Wolbachia* was equally detected in all mosquitoes tested (100%) regardless of the laboratory rearing generation (Fs) (data not shown). Genomic DNA extracted from a *Wolbachia*-free native strain of *Ae. aegypti*was used as negative control of the assay. Data shows that mosquito rearing conditions preserved *Wolbachia* infection between generations (S1G Fig).

# Cytoplasmic incompatibility (CI)

CI experiments are performed every fourth month (three times per year) as a quality control process of the mass-rearing system. The CI was determined before male releasing (May) and during the pilot trial (August and December) by setting up a series of crossing experiments (3 replicates of 50 mosquitoes) between *Wolbachia*-infected (*w*AlbB *Ae. aegypti*) and wild type non-infected (MID) mosquitoes divided into two experimental groups described as follow: a) CI group, ♀ *w*AlbB *Ae. aegypti* x ♂MID; ♀MID x ♂ *w*AlbB *Ae. aegypti* and b) Control group, ♀ *w*AlbB *Ae. aegypti* x ♂ *w*AlbB *Ae. aegypti*; ♀MID x ♂MID. For all crossing experiments, females were blood fed 7-days after caging and mosquitoes were provided oviposition cups 24 h after blood fed for two straight days; eggs were collected and stored until hatching at 26±1. Hatching rates (HR) were scored after 72 h [9].

Crossing between *Wolbachia*-infected males (*w*AlbB *Ae. aegypti*) and wild type females (MID) mosquitoes presented high levels of unidirectional cytoplasmic incompatibility, which resulted in unviable eggs (here identified as broken or collapsed). In the CI crossing group (♀MID x ♂ *w*AlbB *Ae. aegypti*), 100% of laid eggs collapsed and no hatched. For the group ♀ *w*AlbB *Ae. aegypti* x ♂MID, the hatching rate was 83.7%, and for both control groups including *Wolbachia*-infected mosquitoes (♀*w*AlbB *Ae. aegypti* x ♂ *w*AlbB *Ae. aegypti*) and wild type mosquitoes (♀MID x ♂MID), this hatching rate was 79.28% and 78.66%, respectively (Table B).

# Table B. Cytoplasmic incompatibility (CI) from the different mating crosses.

| **Group** | **Cross-mating (3 rep.)** | **Total eggs** | **Unviable eggs collapsed/**  **broken** | **Mean No.**  **eggs/ 50 females** | **Mean No. hatched eggs** | **Mean egg hatch rate** |
| --- | --- | --- | --- | --- | --- | --- |
| CI group | ♀MID (50)  ♂ *w*AlbB *Ae. aegypti* (50) | 1574 | 1549 | 31.48 | 0 | 0 |
|  | ♀ *w*AlbB *Ae. aegypti* (50)  ♂MID (50) | 1409 | 100 | 28.18 | 347.67 | 83.70% |
| Control group | ♀ *w*AlbB *Ae. aegypti* (50)  ♂ *w*AlbB *Ae. aegypti* (50) | 2847 | 166 | 56.94 | 713 | 79.28% |
|  | ♀MID (50) ♂MID (50) | 2181 | 122 | 43.62 | 815.50 | 78.66% |

**Irradiation of *Wolbachia*-infected *Aedes aegypti* on female sterility**

Pupae were collected in 2-h to assure uniform pupal age between 24-30 h. Pupae were sexed based on pupal size dimorphism using a Wolbaki sex sorter and verified under a stereomicroscope. A sample of 500 female and 500 male pupae were placed in 12x12 cm petri dishes (one dish per sex) and irradiated at 45 Gy [2]. Following irradiation, males and females were placed in a 30 × 30 × 30 cm BugDorm-1 cages for emergence with 1:1 (50:50) ratio as follows: ♂ir- *w*AlbB X ♀ir- *w*AlbB, ♂ir- *w*AlbB X ♀nir- *w*AlbB y ♂nir- *w*AlbB X ♀nir- *w*AlbB (Table C). Adult mosquitoes were maintained with 10% sugar solution and allowed to mate for 5 days. All batches were blood fed and 24-h later oviposition cups containing ovistrips and water were set in each cage for egg collection. Ovistrips containing eggs were collected at 48 h and slowly dried for 2 days and stored for 2 days at room temperature before hatching. The total number of eggs per ovistrip were counted and viable eggs (verified using a stereomicroscope) were hatched, and the hatching rate was calculated at 48 h.

**Table C**. Irradiation treatments on male and female *Wolbachia*-infected *Ae. aegypti*. Radiation dose of 45 Gy was applied and crosses between irradiated and non-irradiated mosquitoes and the effect on egg hatching are shown.

| **Experimental group** | **Replicates** | **Total, eggs** | **Hatching eggs** | **Eggs hatch %** |
| --- | --- | --- | --- | --- |
| ♂ir- *w*AlbB X ♀ir- *w*AlbB | 2 | 0 | 0 | 0 |
| ♂ir- *w*AlbB X ♀nir- *w*AlbB | 2 | 804 | 407 | 51 |
| ♂nir- *w*AlbB X ♀nir- *w*AlbB | 2 | 934 | 628 | 67 |

# Male competitiveness of irradiated *Wolbachia*-infected *Aedes aegypti*

The experiments are conducted every fourth month (three times per year) as a quality control process of the mass-rearing system. During the pilot trial, (August and December), an experiment with three replicates was conducted to determine mating competitiveness of irradiated *Wolbachia*-infected *Ae. aegypti* males after being irradiated at 45 Gy. The mating ratios between irradiated *Wolbachia*-infected males (♂ir- *w*AlbB *Ae. aegypti)* vs non-irradiated wild type males (♂nir-MID) and non-irradiated wild type females (♀nir- MID) were 1:1:1. Batches of 50 ♀nir- MID, 50 ♂nir- MID and 50 ♂ir- *w*AlbB *Ae. aegypti* were introduced into each cage (16 cm x 16 cm x 16 cm) respectively. The number of replicates for each mating were two. The experimental groups were left under insectary at 80 ± 5% humidity, 26 ± 1 ̊C temperature, and a photoperiod of L12:D12. A 10% sugar solution was provided as a food source for adult mosquitoes, and cattle blood was provided for females a few days after male introduction. The oviposition cups were introduced to each mosquito cage to collect eggs. After a few days, the oviposition cup with each egg paper was collected, dried at room temperature, and then the eggs were counted before hatching in deionized water. The total number of eggs hatched and egg hatch rate from each egg batch were recorded at 48 h to determine the male mating competitiveness. The male mating competitiveness index (C) was calculated as: C = [(Hn—Ho) / (Ho—Hs)] * (N / S); where Hn is the hatching rate of the fertile control ♀nir-MID x ♂nir-MID eggs; Ho is the hatching rate of competition group ♀nir-MID x ♂nir-MID x ♂ir- *w*AlbB *Ae. aegypti* eggs; Hs is the hatch rate of the sterile controls ♀MID x ♂ir- *w*AlbB *Ae. aegypti* and N and S are the numbers of fertile and sterile males, respectively [10]. The induced sterility (IS) value was calculated as 100% minus the residual fertility value, which was calculated from Ho /Hn [11].

The cross mating 1: 1: 1 showed a hatching rate of 36.16 %. Meanwhile, the hatching rate of control (fertile) was 83.03% and the second control (infertile) no eggs hatched (Table D). The residual fertility value obtained was 0.43. The competitiveness index (C) compared with control was 1.29 meaning that *Wolbachia*-infected males (sterile) were fully competitive that uninfected males. The residual fertility was 0.43 and induced sterility (IS) was 99.57%.

# Table D. Competitiveness between *Wolbachia*-infected males (*w*AlbB *Ae. aegypti*) and unirradiated wild type males (MID). Mean (± SD) are shown.

| **Experimental group** | **Total eggs** | **Unviable eggs** | **Total hatched eggs** | **Eggs hatch rate** |
| --- | --- | --- | --- | --- |
| Competitiveness group | 2,052 (1.026+28) | 725  (362±133) | 1,327  (663±161) | 36.16% |
| Fertile control group | 1,799  (899±48) | 79  (39±0.70) | 1, 720  (869±48) | 83.03% |
| Sterile control group | 723  (361±27) | 723  (361±27) | 0 | 0 |

**Table E** . Percentage of *Ae. albopictus* larvae hatched per individual ovistrip (No. *Ae. albopictus* larvae/Total *Aedes* eggs x 100) at San Pedro Chimay (SPC) and Tahdzibichén (TAH).

| **Site/Week** | **39** | **40** | **41** | **42** | **43** | **44** | **45** |
| --- | --- | --- | --- | --- | --- | --- | --- |
| SPC | 33.4% | 56.2% | 40.0% | 34.0% | 6.6% | 24.9% | 16.7% |
| TAH | 21.3% | 31.2% | 22.9% | 9.3% | 3.4% | 12.0% | 12.8% |
| **Average** | **28.0%** | **45.1%** | **33.6%** | **24.7%** | **5.1%** | **20.1%** | **15.4%** |

| **Site/Week** | **46** | **47** | **48** | **49** | **50** | **51** | **Average** |
| --- | --- | --- | --- | --- | --- | --- | --- |
| SPC | 0.0% | 16.0% | 35.7% | 40.0% | 12.8% | 32.1% | 29.2% |
| TAH | 12.8% | 4.6% | 0.0% | 9.2% | 1.1% | 0.0% | 12.5% |
| **Average** | **9.6%** | **10.3%** | **10.2%** | **27.3%** | **8.6%** | **25.0%** | **22.1%** |

# References

1. Carvalho DO, Nimmo D, Naish N, McKemey AR, Gray P, Wilke AB, et al. Mass production of genetically modified *Aedes aegypti* for field releases in Brazil. JoVE. 2014;83: e3579. doi: 10.3791/3579.
2. Zheng X, Zhang D, Li Y, Yang C, Wu Y, Liang X, et al. Incompatible and sterile insect techniques combined eliminate mosquitoes. Nature. 2019;572: 56-61. doi: <https://doi.org/10.1038/s41586-019-1407-9>.
3. Zhang D, Lees RS, Xi Z, Bourtzis K, Gilles JRL. Combining the Sterile Insect Technique with the Incompatible Insect Technique: III-Robust Mating Competitiveness of Irradiated Triple Wolbachia-Infected Aedes albopictus Males under Semi-Field Conditions. PLoS One. 2016; 11(3): e0151864. doi:https://doi.org/10.1371/journal.pone.0151864.
4. Bourtzis K, Robinson AS. Insect pest control using *Wolbachia* and/or radiation. In: Bourtzis K, Miller TA editors. Florida: Insect symb. 2006;2: 225-246.
5. Bourtzis K, Dobson SL, Xi Z, Rasgon JL, Calvitti M, Moreira, et al. Harnessing mosquito–*Wolbachia* symbiosis for vector and disease control. Acta Trop. 2014;132 Suppl: S150-S163. doi: 10.1016/j.actatropica.2013.11.004.
6. Kamtchum-Tatuene J, Makepeace BL, Benjamin L, Baylis M, Solomon T. The potential role of *Wolbachia* in controlling the transmission of emerging human arboviral infections. Current opinion in infec Dis. 2017;30(1): 108-116. doi: 10.1097/QCO.0000000000000342.
7. Timmermann SE, Briegel H. Molting and metamorphosis in mosquito larvae: a morphometric analysis. Mitt Schweiz Entomol Ges (Print). 1998;71: 373-387. doi: <http://doi.org/10.5169/seals-402722>.
8. Puerta-Guardo H, Contreras-Perera Y, Pérez-Carrillo S, Che-Mendoza A, Ayora-Talavera G, Vazquez-Prokopec G. et al. *Wolbachia* in Native Populations of *Aedes albopictus* (Diptera: Culicidae) From Yucatan Peninsula, Mexico. J Insect Sci. 2020;20(5): 16. doi: 10.1093/jisesa/ieaa096.
9. Zheng ML, Zhang DJ, Damiens DD, Lees RS, Gilles JRL. Standard operating procedures for standardized mass rearing of the dengue and chikungunya vectors *Aedes aegypti* and *Aedes albopictus* (Diptera: Culicidae) - II - Egg storage and hatching. Parasites Vectors. 2015;8: 348. doi: https://doi.org/10.1186/s13071-015-0951-x.
10. Fried M. Determination of sterile-insect competitiveness. J Med Entomol. 1971;64(4): 869-872. doi: <https://doi.org/10.1093/jee/64.4.869>.
11. Yamada H, Vreysen MJ, Gilles JR, Munhenga G, Damiens DD. The effects of genetic manipulation, dieldrin treatment and irradiation on the mating competitiveness of male *Anopheles arabiensis* in field cages. Malar J. 2014;13: 318. doi: 10.1186/1475-2875-13-318.
