# Supplementary figures and images for: Pilot trial using mass field-releases of sterile males produced with the incompatible and sterile insect techniques as part of integrated Aedes aegypti control in Mexico
Source: PLoS Negl Trop Dis. 2022 Apr 26;16(4):e0010324. doi: 10.1371/journal.pntd.0010324 (PMC9041844; doi:10.1371/journal.pntd.0010324)

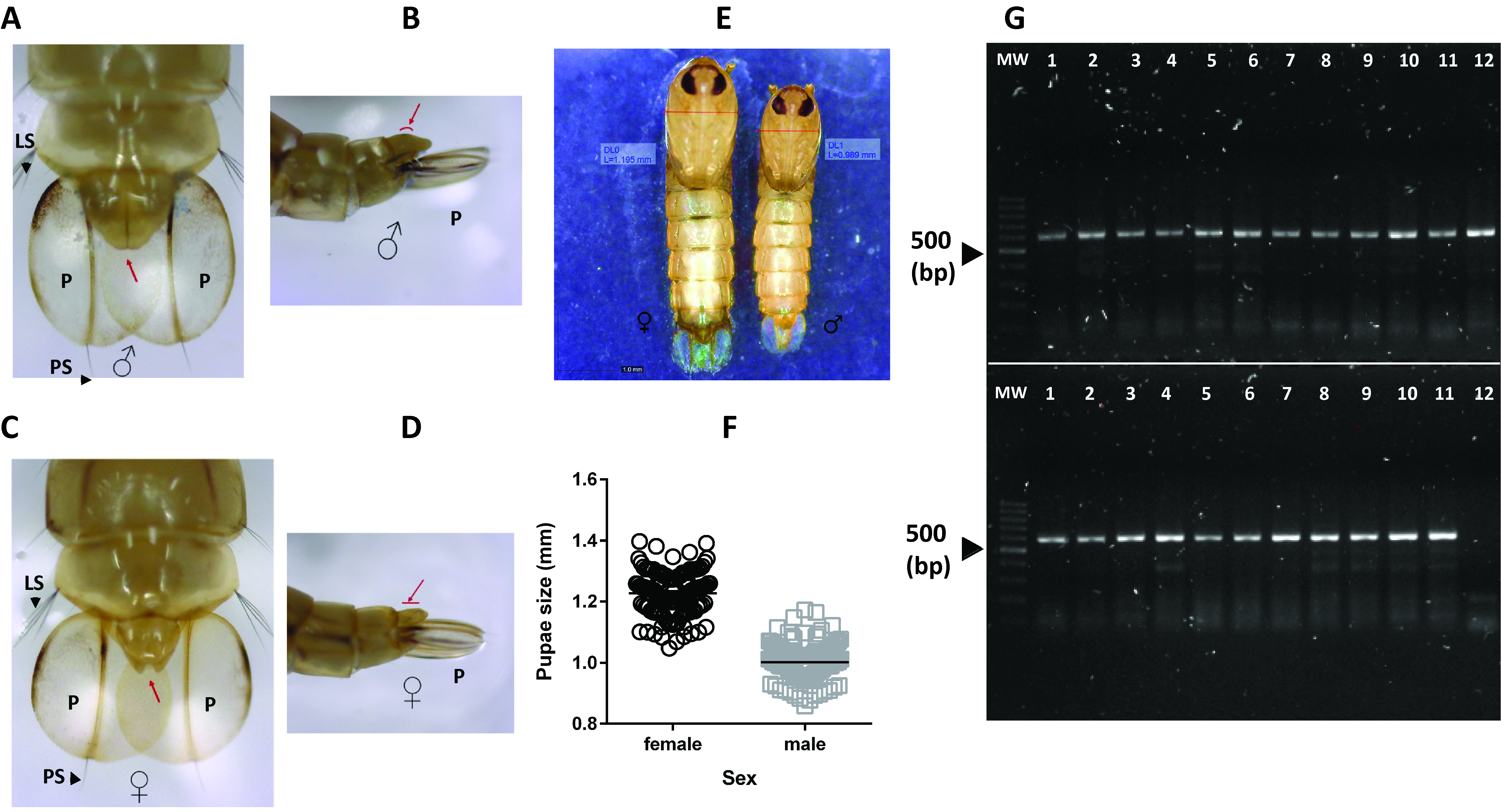

Supplement: S1 Fig — (A, C) ventral and lateral (B, D) views of pupal paddles, showing dimorphism characters between males (A, B) and females (C, D). Red arrows indicate the location and morphology of the genital lobes in male and female Ae. aegypti mosquitoes. Abbreviations: P, paddle; PS, paddle seta; LS, lateral seta. Pupae sizes of male and female Ae. aegypti mosquitoes. (E) Representative images of male and female pupae (Magnification: 50X). Ventral view of female (left) and male (right) pupae. Red lines show the diameter (mm) of the cephalothorax in the ventral position. (F) Pupae size diameter (mm) of female (n = 125) and male (n = 125) mosquitoes (F11, -13, -16, -18). Data represents the media plus-minus the standard error. (G) Detection of Wolbachia genome in male Ae. aegypti mosquitoes reared under laboratory conditions. Total genomic DNA was extracted from two different generations (F19, n = 29, F30, n = 29) of laboratory-reared adult male Ae. aegypti mosquitoes artificially infected with Wolbachia stain B. Representative image of Wolbachia infection detected in male mosquitoes obtained from generations F19 (n = 11) and F20 (n = 11). Genomic DNA from a female Ae. aegypti (F6) artificially infected with Wolbachia strain B (wAlbB Ae. aegypti) (lane 12, top image), and a Wolbachia free native Ae. aegypti from Yucatan (lane 12, lower image) were used as positive and negative controls of the assay, respectively. DNA marker (100 bp). Agarose gel (1%) stained with SYBR safe. PCR positive amplicon: ~600 bp. (TIF) [file pntd.0010324.s003.tif]
